# Optimization of Fly Ash—Slag One-Part Geopolymers with Improved Properties

**DOI:** 10.3390/ma16062348

**Published:** 2023-03-15

**Authors:** Iman Faridmehr, Mohammad Ali Sahraei, Moncef L. Nehdi, Kiyanets A. Valerievich

**Affiliations:** 1 Department of Building Construction and Structural Theory, South Ural State University, 76 pr. Lenina, 454080 Chelyabinsk, Russia; 2 Department of Civil Engineering, College of Engineering, University of Buraimi, Al Buraimi 512, Oman; 3 Department of Civil Engineering, McMaster University, Hamilton, ON L8S 4M6, Canada

**Keywords:** one-part geopolymer concrete, compressive strength, artificial neural network, alkali-activated material, industrial by-product

## Abstract

One-part geopolymer concrete/mortar is a pre-mixed material made from industrial by-products and solid alkaline activators that only requires the addition of water for activation. Apart from being environmentally friendly, it also reduces complexity and improves consistency in the mixing process, leading to more efficient production and consistent material properties. However, developing one-part geopolymer concrete with desirable compressive strength is challenging because of the complexity of the chemical reaction involved, the variability of the raw materials used, and the need for precise control of curing conditions. Therefore, 80 different one-part geopolymer mixtures were compiled from the open literature in this study, and the effects of the constituent materials, the dosage of alkaline activators, curing condition, and water/binder ratio on the 28-day compressive strength of one-part geopolymer paste were examined in detail. An ANN model with the Levenberg–Marquardt algorithm was developed to estimate one-part geopolymer’s compressive strength and its sensitivity to binder constituents and alkaline dosage. The ANN model’s weights and biases were also used to develop a CPLEX-based optimization method for achieving maximum compressive strength. The results confirm that the compressive strength of one-part geopolymer pastes increased by increasing the Na_2_O content of the alkaline source and the slag dosage; however, increasing the Na_2_O content in alkaline sources beyond 6% by fly ash weight led to decreasing the compressive strength; therefore, the optimum alkaline activator dosage by weight of fly ash was to be 12% (i.e., 6% Na_2_O). The proposed ANN model developed in this study can aid in the production and performance tuning of sustainable one-part geopolymer concrete and mortar for broader full-scale applications.

## 1. Introduction

Concrete is the world’s second most consumed commodity after water and the most used construction material globally. This results in a colossal environmental footprint with considerable carbon emissions and depletion of natural resources. Around 8% of all CO_2_ emissions worldwide are related to concrete, and most of those emissions come from the manufacture of cement [1]. According to some estimates, 4.2 billion tons of cement are produced annually worldwide, causing about 4 billion tons of CO_2_ emission into the atmosphere [2]. The manufacture of one ton of ordinary Portland cement (OPC) emits about 0.8 to 1 ton of CO_2_. Thus, there is growing pressure on the concrete industry to develop different binders to reduce the need for OPC. To produce environmentally friendly concrete, it is necessary to develop viable alternatives to OPC that emit little or no CO_2_ [3,4].

One of the potential options to lessen the environmental impact of OPC binders is the development of low-carbon binders [5,6]. Aluminosilicate materials react with water slowly, but when exposed to hydrolysis and condensation reactions in an alkaline solution, they react more quickly to produce inorganic polymers that can withstand mechanical loads. The structure of aluminosilicates significantly impacts the binding behavior that results from the amorphous aluminosilicate gels and the reactivity of the source materials, and numerous studies have been devoted to this issue [7].

The synthesis of aluminosilicates using alkalis can be categorized into low-calcium aluminosilicates and high-calcium aluminosilicates, depending on the calcium content of the source materials. Low calcium content aluminosilicate materials produce a sodium aluminosilicate hydrate (N-A-S-H), whereas high calcium content aluminosilicate materials produce calcium aluminosilicate hydrate (C-A-S-H), which resembles Portland cement C-S-H. Geopolymer binders are environmentally friendly materials used as a substitute for OPC binders. The polymerization mechanism of geopolymers is an intensely rapid chemical reaction of silica-alumina minerals in an alkaline environment that yields a three-dimensional polymeric sequence and ring structure consisting of Si–O–Al–O bonds. Geopolymer concrete production does not require the use of OPC, but the reaction of an aluminosilicate ingredient with strong alkaline liquids can produce a binder.

A novel method called one-part alkali-activation has been created to simplify the handling of traditional geopolymers, which involves mixing aluminosilicate precursors with powdered activators instead of an alkaline solution [8,9]. In contrast to traditional geopolymer binders, where solutions are used as the activation phase, the activators in the one-part binders are in dry powder form, and the reaction begins as soon as water is added to the binder. This method avoids producing geopolymer concrete in large quantities using corrosive and caustic solutions. One-part geopolymers emit less carbon dioxide into the atmosphere than conventional geopolymers because only a small portion of the framework is formed during the polymerization process [10]. Similar to OPC concrete preparation, one-part alkali-activated binders are made by adding water to a dry mixture comprising a solid aluminosilicate precursor and a solid alkali activator. Compared to OPC mixtures, manufacturing one-part geopolymers increases the geopolymer’s economic viability and the potential to curb CO_2_ emissions significantly.

A computational model based on an artificial neural network (ANN) mimics the biological neural networks in the brain to process information. Neural networks can “learn” and correlate massive datasets gleaned from simulations or experiments. The trained neural network can be used as an analytical tool to make accurate predictions about problem outcomes. They can produce excellent prediction accuracy with practical approaches for training and validation. Hence, this paper uses ANNs to estimate the compressive strength of one-part geopolymer pastes. An experimental database was compiled from information available in the open literature and used to train the ANN models. Five input parameters were used to train the ANN model: constituent materials, alkaline source content, curing temperature, and water/binder ratio. A “trial-and-error” approach was used to determine the input parameter weights that would produce the most accurate prediction of compressive strength. The best effective pattern for predicting compressive strength during the training approach was found by the Levenberg–Marquardt training (LM).

While several studies have reported on the effectiveness of one-part alkali-activated materials [11,12,13], the effects of the binder constituent, alkaline source content, curing condition, and water/binder ratio on the 28-day compressive strength have not yet been thoroughly investigated. This study examined the effects of the input parameters by compiling 80 distinct fly ash/GGBS-based geopolymer pastes from the open literature. An informative model employing the Levenberg–Marquardt algorithm was developed with the help of the prepared database to estimate the compressive strength and its sensitivity to the input parameters. Utilizing the weights and biases of the ANN model, a CPLEX-based optimization method was developed to determine the ideal combination of parameters for attaining the highest compressive strength. The “ideal” mixture of one-part geopolymer paste might replace OPC binders and be used to create geopolymer concrete and mortar. Additionally, the proposed binder can be ideal for underwater construction, repair, and maintenance applications. Using the proposed informational model, users would have an insight into the influential parameters on 28-day compressive strength, and also, with the aid of the CPLEX-based optimization method, they can optimize the input parameters to achieve the maximum compressive strength. The objective is to enhance the prediction accuracy of the user-friendly one-part geopolymer, which would make it more convenient for application in diverse building projects, particularly in harsher settings like coastal areas.

## 2. Literature Review

The conventional method of using an alkaline activator solution in geopolymer production involves the use of highly caustic sodium- or potassium-based hydroxide, silicates, carbonates, or their combinations. This makes it dangerous to handle, store, and transport, requiring additional safety precautions that can slow down production and increase costs. To overcome these challenges, researchers have explored the use of solid activators to produce a user-friendly one-part geopolymer that only requires the addition of water. Some of the notable materials used as alkali sources include sodium hydroxide combined with various silica sources such as fly ash, rice husk ash, micro silica, calcium hydroxide, different grades of sodium metasilicate, and red mud. In recent years, many studies have focused on investigating the mechanical properties and durability of one-part geopolymer mortars and concrete.

In their study, Askarian and colleagues [8] created one-part hybrid concrete mixes using a combination of ordinary Portland cement (OPC) and geopolymers. They added solid potassium carbonate, which made up 7.5% of the total geopolymeric raw materials, as the primary activator. The researchers blended fly ash and ground granulated blast-furnace slag with the geopolymeric raw materials in various proportions and found that the addition of OPC decreased workability and setting time. However, it notably enhanced early age and ultimate compressive strength due to the rapid reaction of OPC with alkali activators.

Muthukrishnan et al. [14] conducted a study on the rheochemical approach to analyze the early strength development resulting from alkali reactions and formulate a suitable 3D printable one-part geopolymer concrete. The researchers evaluated the impact of different design parameters, such as activator content, thixotropic additive (Magnesium Alumino Silicate—MAS), and retarder (sucrose) dosage, on the rheological properties of the concrete. The findings indicated that the one-part geopolymer formulation exhibited improved printing characteristics when the binder contained 0.75 wt% MAS, 10 wt% activator, and 1.5 wt% sucrose.

Muhammad Riaz Ahmad et al. developed a new type of energy-efficient and sustainable concrete based on industrial waste materials and vegetal aggregate for hygrothermal and low load-bearing applications. They conclude that the vegetal concrete mixtures containing red mud exhibited higher capillary and water absorption as compared to other mixtures. Moreover, all concrete mixtures were classified as good to excellent moisture buffer materials.

Dongthe et al. [15] studied the solid activator, the synthetic sodium metasilicate pentahydrate against water, and a hybrid sodium silicate and sodium hydroxide activator solution to develop a high-strength one-part geopolymer mortar. They conclude that the solid activator using sodium silicate pentahydrate outperformed the often-used liquid activator in terms of the compressive strength of the mortar. Nevertheless, the compressive strength decreased, and efflorescence increased significantly once the metasilicate content exceeded Na_2_O% = 6%.

Wangthe et al. [16] investigated the early-age properties of one-part fly ash/ground granulated blast-furnace slag (FA/GGBS) geopolymer through the utilization of hybrid activators, such as anhydrous sodium metasilicate (Na_2_SiO_3_), sodium carbonate (Na_2_CO_3_), and sodium aluminate (NaAlO_2_). They indicated that Na_2_SiO_3_-activated one-part geopolymer released high reaction heat and achieved a faster setting. Such shortcomings could be improved by partially replacing Na_2_SiO_3_ with Na_2_CO_3_ in a solid form. Besides, incorporating slight NaAlO_2_ decreased the self-flow of geopolymer paste, whereas the slump-flow properties remained unchanged.

The previous research mainly acknowledged that to meet the dual requirements of convenience and eco-friendliness in practical engineering, the synthesis of one-part fly FA/GGBS geopolymer binders’ selection and content of different hybrid combinations of solid activators, including anhydrous Na_2_SiO_3_, Na_2_CO_3_, and NaAlO_2_, etc. is essential. Flowability, setting time, strength, and effect of one-part geopolymer paste molar ratios at different activator dosages were examined to determine the fundamental properties of one-part geopolymer paste cured in the ambient.

## 3. Materials and Methods

### 3.1. Database Description

The most comprehensive 28-day compressive strength data for various one-part geopolymer pastes was collected from accessible, pertinent data in the open literature [17,18,19,20,21,22,23,24,25,26,27,28]. The database comprises 80 FA/GGBS binder-based one-part geopolymers made with an anhydrous sodium silicate alkaline source, see Table 1.

The source publications investigated the effects of different parameters on the compressive strength of one-part geopolymers, including the percentage of fly ash and GGBS and the Na_2_O dosage of the alkaline source. Although the variation of water/binder ratio and curing temperature was not considerable, their effects on compressive strength were examined. The range of input/output parameters of the studied dataset is shown in Table 2.

To activate the binder components in fly ash/GGBS-based geopolymers, granular sodium metasilicate (Na_2_SiO_3_) anhydrous (50% Na_2_O, 46% SiO_2_, and 4% H_2_O) is often employed as a solid activator. Since, unlike two-part geopolymers, there is no need to create a NaOH solution before mixing, using granular alkaline activators in one-part geopolymer systems is more straightforward and faster than using the commonly used and caustic alkaline solutions. The granular sodium metasilicate anhydrous was used at 3 to 25% by weight of the binding materials in the studied database.

In the prepared database, pure GGBS was used without further treatment as the main resource of calcium materials in geopolymer production. Low calcium fly ash was also used as another source of precursor materials. Fly ash and GGBS are used in geopolymer concrete because they can react with an alkaline activator to form a binder that can replace Portland cement. Geopolymer concrete made with these materials has several advantages over traditional concrete, including higher strength, lower permeability, and better resistance to chemical attack [29]. GGBS also contains silicates and alumina, which are necessary for the formation of geopolymer binders, and its high specific gravity can increase the density of the concrete and make it more resistant to erosion. Additionally, the use of fly ash and GGBS in geopolymer concrete is a sustainable and environmentally friendly approach to construction that reduces waste and produces a high-quality, durable material. The chemical components of the fly ash and GGBS (generally available in the market) were analyzed by studied references using X-Ray fluorescence (XRF), see Table 3.

### 3.2. Mixing Procedure and Test Methods

**Figure 1:**
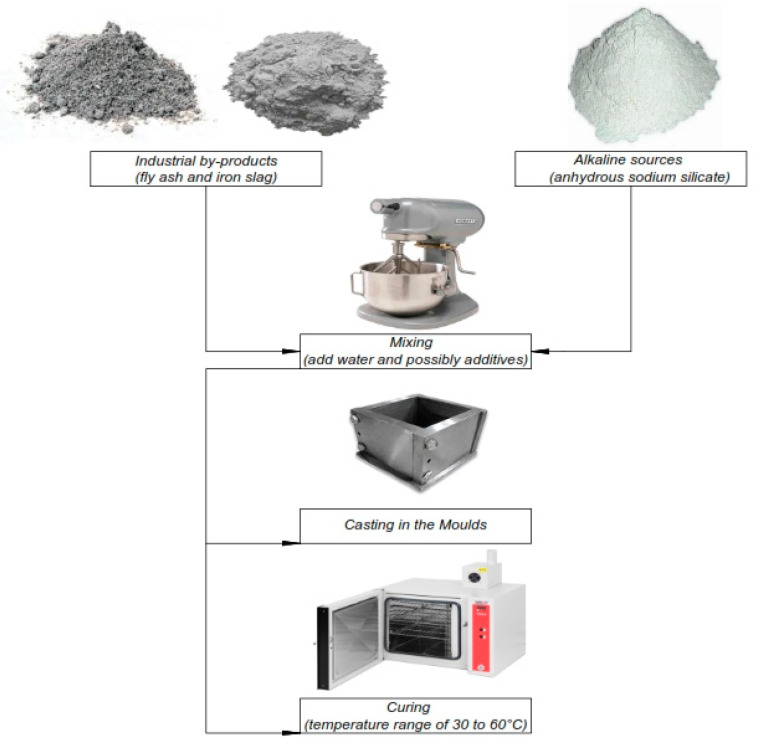
Block diagram for one-part geopolymer paste production.

Preparing one-part geopolymer paste followed the ASTM C305-14 [30] recommended procedures. Table 1 lists the 80 various geopolymer pastes prepared with fly ash and GGBS precursor materials and various Na_2_O content of alkaline sources. Based on these experimental tests, the granular sodium metasilicate anhydrous and binding materials were mixed for about two minutes using a mechanical mixer. After adding potable tap water to the dry mixture, mixing resumed for an additional three minutes to achieve homogeneity and consistency. Figure 1 shows the block diagram for one-part geopolymer paste production. The studied literature also acknowledged that the compressive strength tests were performed following the guidelines of the ASTM C109-109M [31] standards.

## 4. Compressive Strength Results and Discussion

**Figure 2:**
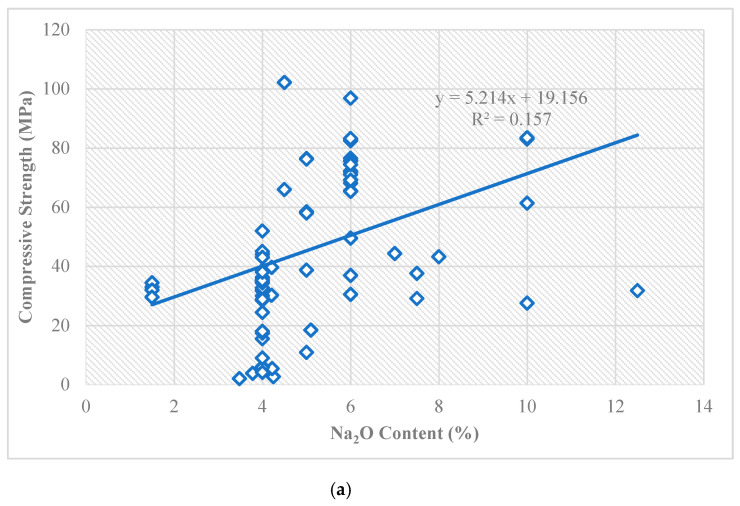

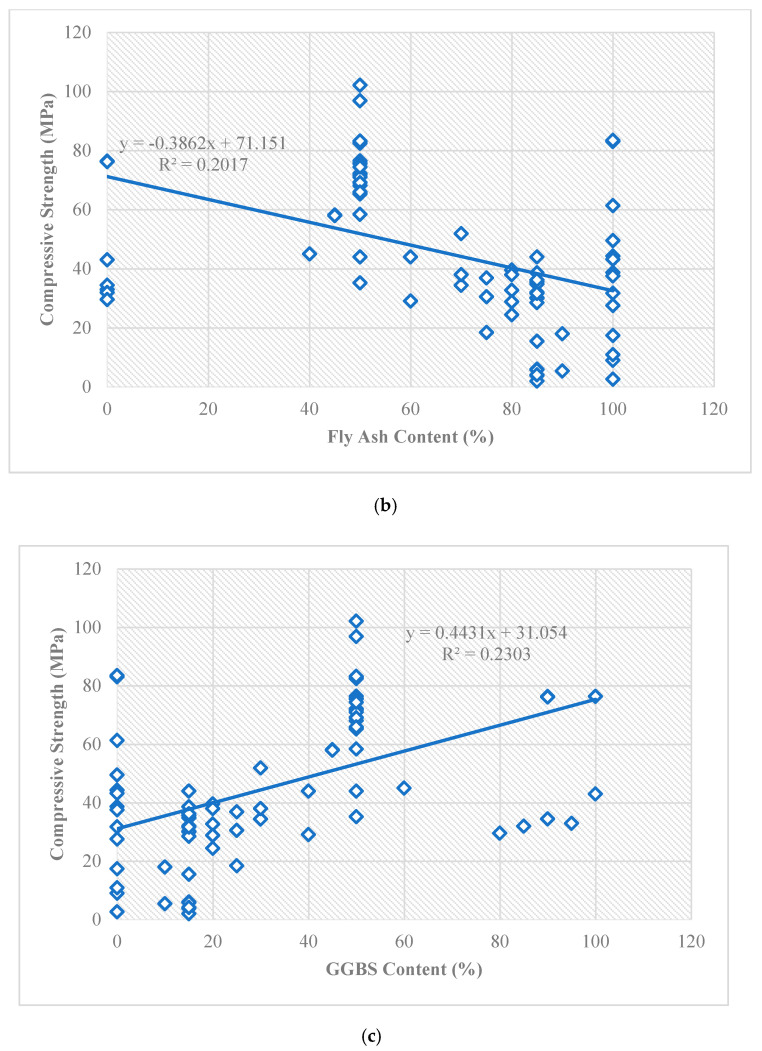
Effects of (**a**) Na_2_O content, (**b**) fly ash content, and (**c**) GGBS content on compressive strength of one-part geopolymer paste.

The 28-day compressive strength results of all studied mixture designs are presented in Table 1. The effects of the Na_2_O, fly ash, and GGBS contents on the compressive strength of one-part geopolymer paste are depicted in Figure 2. The results indicate that increasing the GGBS and Ca_2_O contents increased the compressive strength of the one-part geopolymer paste. Lower Ca_2_O content led to insufficient alkali and negatively impacted the system’s geopolymerization process. The result indicates that raising the granular alkaline activator content by weight of the fly ash beyond 12% slightly decreased the compressive strength and workability of the one-part geopolymer cement pastes [9]. The optimum alkaline activator dosage by weight of fly ash was found to be 12% (i.e., 6% Na_2_O) to attain the highest compressive strength at 28 days of curing.

However, the compressive strength of one-part geopolymer paste was negatively affected by the fly ash content. Prior studies have established that geopolymer concrete produced with class C fly ash, which has a high calcium concentration, exhibits higher compressive strength at ambient temperatures than geopolymer concrete made with class F fly ash [32]. Because geopolymer concrete produced with class F fly ash cannot achieve structural integrity at room temperature, it is normally heat cured to 60 °C. At high temperatures, geopolymer concrete produced with class F fly ash outperforms geopolymer concrete manufactured with class C fly ash in terms of mechanical strength. In addition to the calcium content, the particle size distribution, specific surface area, and chemical composition of fly ash can also affect the performance of geopolymer concrete. For instance, fly ash with a higher specific surface area can result in higher compressive strength due to the increased reactivity and better distribution of the geopolymer precursors. Moreover, the chemical composition of fly ash can vary depending on the source and type of coal used, which can influence the geopolymerization reaction and the resulting properties of the geopolymer concrete. Therefore, careful selection and characterization of fly ash is critical to ensure the desired performance and consistency of geopolymer concrete.

The low correlation coefficients in Figure 2 acknowledge that the investigated variables interact with each other in a complex way and therefore is difficult to capture this relationship with a simple regression equation. In such cases, alternative statistical methods may be more appropriate for modeling the relationship between the investigated variables and 28-day compressive strength. For example, non-linear regression models or machine learning algorithms, such as decision trees or neural networks, could potentially capture the non-linear relationship and underlining mechanism between these variables.

## 5. Artificial Intelligence Estimation of Compressive Strength

### 5.1. Artificial Neural Networks

Researchers are increasingly using artificial intelligence approaches such as genetic algorithms, adaptive regressions, fuzzy logic, and artificial neural networks (ANNs) instead of traditional or classical methods such as linear regression, time-series analysis, etc. [33,34,35]. While these classical methods have been widely used and are still valuable in certain contexts, artificial neural networks (ANNs) have shown to be more powerful in modeling complex non-linear relationships and are, therefore, increasingly being used in many fields, including image recognition, natural language processing, and financial modeling. ANNs are a type of machine learning method that draws inspiration from the structure and operation of the human brain. ANNs are composed of interconnected nodes or neurons that carry out information processing in parallel. ANNs are typically used when the relationship between the input and output is complex or when using another available approach requires considerable investment in time and money. In feed-forward networks, one of the most often used types of ANNs, neurons are arranged in layers containing an input layer, one or more hidden layers, and an output layer. Using network weights and biases, neurons in the hidden layer are linked to those in the preceding and following layers. Moreover, to reduce prediction errors, ANNs should be trained using an optimization approach in which the training function would modify the network weights matrix for each epoch.

The backpropagation (BP) learning algorithm has been successfully utilized with various numerical optimization approaches to accelerate network convergence. Combining the Levenberg–Marquardt, a common non-linear least squares optimization algorithm, into the BP algorithm was proven to be highly effective. The Levenberg–Marquardt has higher convergence, generalization, and precision than other algorithms, and fewer iterations (epochs) are needed to attain lower error rates [34]. Despite being a quick and effective optimization technique, the Levenberg–Marquardt approach has the limitation that it could be trapped in a local minimum [36].

### 5.2. Generation of Training Model and Statistical Metrics

**Figure 3:**
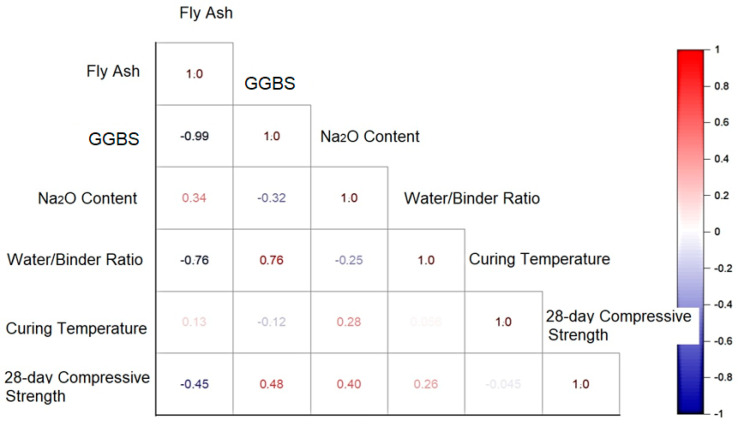
Correlation of input/output parameters.

Correlation or dependency denotes any mathematical relationship, regardless of causation, between two random variables. Correlation measures the strength of the linear association between two variables. A correlation matrix is a tabular representation of the correlation coefficients between the input variables, displaying the relationship between each pair of variables in a table cell. A correlation matrix is useful for summarizing input data for further analysis. Figure 3 illustrates the correlation matrix of the input/output parameters utilized in the present study. Equation (1) was used to normalize each parameter in the range of 1 to −1 while considering each input parameter’s domain and preventing any divergence in the results. *X_n_* is the normalized value of the parameter, where *X_max_* is its maximum value, and *X_min_* is its minimum value. *X* is the variable’s original (non-transformed) value. Table 2 provides the maximum and minimum values for each input parameter. The results of the correlation matrix acknowledge that GGBS and the Na_2_O contents had significant effects on the 28-d compressive strength.

(1) $$X_{n}=\frac{2\left(X-X_{min}\right)}{X_{max}-X_{min}}-1$$

In all the networks created in this study, the hyperbolic tangent function and the Levenberg–Marquardt training algorithm were employed. Following the Kolmogorov technique [37], if the system has a wide enough range of neurons, an ANN using the BP algorithm with one or two hidden layers can easily calculate any continuous function to any level of accuracy [38]. In this regard, the *i*th neuron in the network offers a total that collects the bias (b*_i_*) as well as its weighted input (w*_ij_*) to develop (n*_i_*) as network input which is provided in Equation (2). In this equation, b*_i_* is the *i*th neuron bias; p*_j_* is the input vector; w*_ij_* denotes the strength of the connection from the *j*th input to the *i*th neuron.

(2) $$n_{i}=\sum_{j}w_{ij}p_{j}+b_{i}$$

In order to predict the 28-day compressive strength of one-part geopolymer pastes, an artificial neural network (ANN) was developed using the MATLAB ANN toolbox. The network consisted of five neurons in the input layer, one neuron in the output layer representing compressive strength, and two hidden layers containing various numbers of neurons. The feed-forward neural network was trained using the *trainlm* function to create an effective network. The transfer functions used were “*tansig*” or “hyperbolic tangent sigmoid” for the hidden layers and “*purelin*” or “*Linear*” for the output layer.

The optimal number of neurons for the hidden layers was determined through a trial-and-error process by running the MATLAB code iteratively to obtain the best-performing network. The MATLAB code included a nested loop in determining the best number of neurons for both hidden layers. Initially, a range of 4 to 15 neurons for each loop was selected based on the preliminary code running. The input layer of the network consisted of five parameters, and each neuron in the hidden layers received a distribution of these input parameters multiplied by different weights. The initial magnitude of the bias and weights was presumed for the first iteration. The bias magnitude was related to the hidden layer neurons used as inputs to the specific neurons, and the outputs from each neuron were transferred through an activation function. The output layer neuron then received the magnitude multiplied by specific weights, and errors were determined by comparing the actual values with the model outputs. The bias and weights were updated based on the given learning algorithm for the next iteration, and the process continued until the model converged to the desired degree [39].

In this research, the data were divided randomly into three sets: training (70%), testing (15%), and validation (15%). This specific partition was chosen after testing several other options to ensure the network could be trained effectively without overfitting. During the training process, the aim is to minimize the error function by finding an optimal set of weights and biases that produces outputs similar to the desired values. To evaluate the performance of the developed neural network topologies, statistical indices such as Root Mean Squared Error (RMSE), Mean Squared Error (MSE), Mean Absolute Error (MAE), and correlation coefficient R (expressed in Equations (3)–(6)) were utilized, where $\left({\overset{-}{O}}_{i}\right)$ signifies the magnitude of the real values, $\left({\overset{˘}{y}}_{i}\right)$ is the predicted value of the model, (*O_i_*) signifies the real data, and (*n*) denotes the quantity of observed data.

(3) $$R^{2}=1-\frac{\sum_{i}{\left({\overset{˘}{y}}_{i}-O_{i}\right)}^{2}}{\sum_{i}\left(O_{i}-{\overset{-}{O}}_{i}\right)}$$

(4) $$RMSE=\sqrt{\sum_{i=1}^{n}\frac{{\left({\overset{˘}{y}}_{i}-O_{i}\right)}^{2}}{n}}$$

(5) $$MSE=\frac{1}{n}\sum_{i=1}^{n}{\left({\overset{˘}{y}}_{i}-O_{i}\right)}^{2}$$

(6) $$MAE=\frac{\sum_{i=1}^{n}\left|{\overset{˘}{y}}_{i}-O_{i}\right|}{n}$$

**Figure 4:**
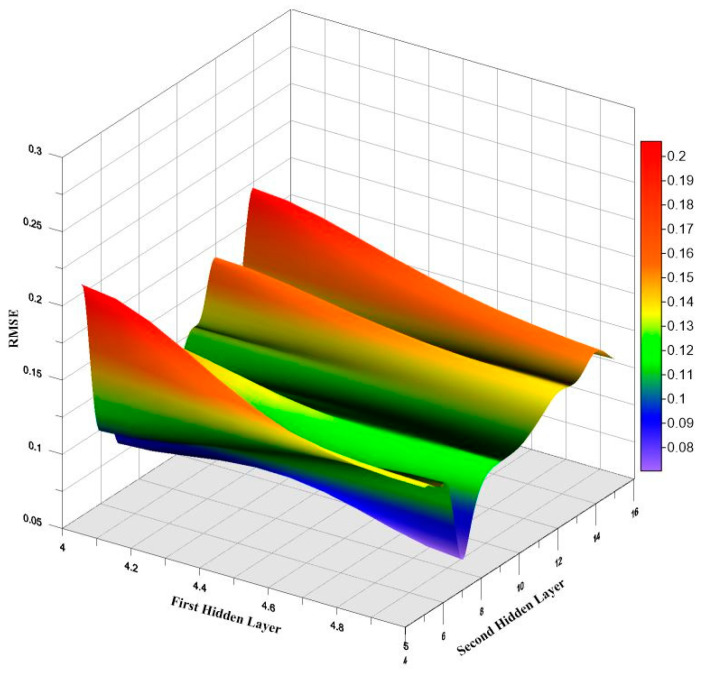
Outcomes of RMSE values based on the *trainlm* function in ANN.

After numerous iterations, the best-performing ANN model was identified as the one using the *trainlm* function in MATLAB with 5 and 7 neurons in the hidden layers. The performance of the developed models was evaluated using statistical indices, including RMSE, MSE, MAE, and correlation coefficient R, with the results shown in Figure 4. From the figure, it is evident that the network with 5 and 7 neurons in the hidden layers yielded the best RMSE values, which were 0.049.

After developing the ANN model, each input variable was assigned a specific coefficient, which was obtained using separate codes from the MATLAB script. Table 4 displays the bias and weights of the optimal model, which was trained using the trainlm algorithm. In an ANN trained with this algorithm, the bias and weights of the optimal model are essential parameters that influence the network’s performance. The trainlm algorithm is a type of backpropagation algorithm that is commonly used for supervised learning tasks such as classification and regression. During training, the algorithm adjusts the weights and biases of the network to minimize the difference between the predicted outputs and the actual outputs for a given set of input data. The optimal values of the weights and biases are those that result in the lowest error or loss on the training data. Once the network is trained, the bias and weights of the optimal model are used to make predictions on new, unseen data. The bias represents the threshold for activation of the neurons in the network, and the weights determine the strength of the connections between the neurons.

**Figure 5:**
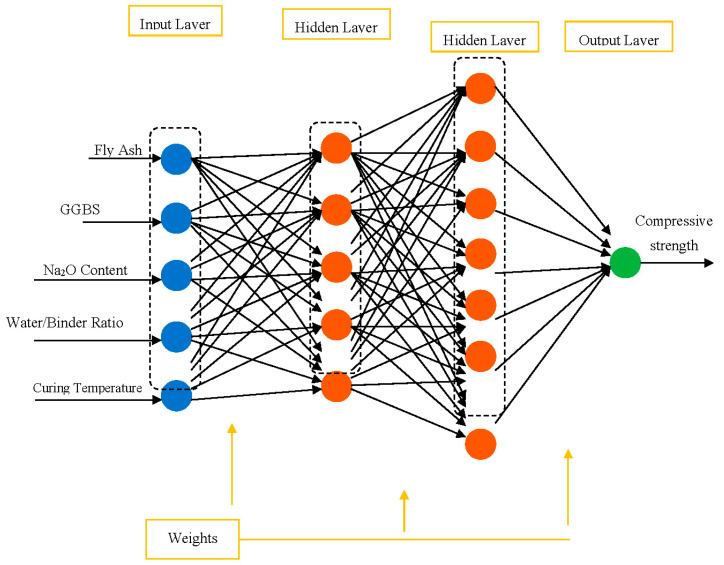
ANN model with optimal topology.

The proposed topology of the feed-forward network with two hidden layers, five input variables (neurons), and one output parameter is depicted in Figure 5. The (*I*) represents the input vector (from *I*1 until *In*), and (*O*) represents the output vector. The lines represent the connections.

**Figure 6:**
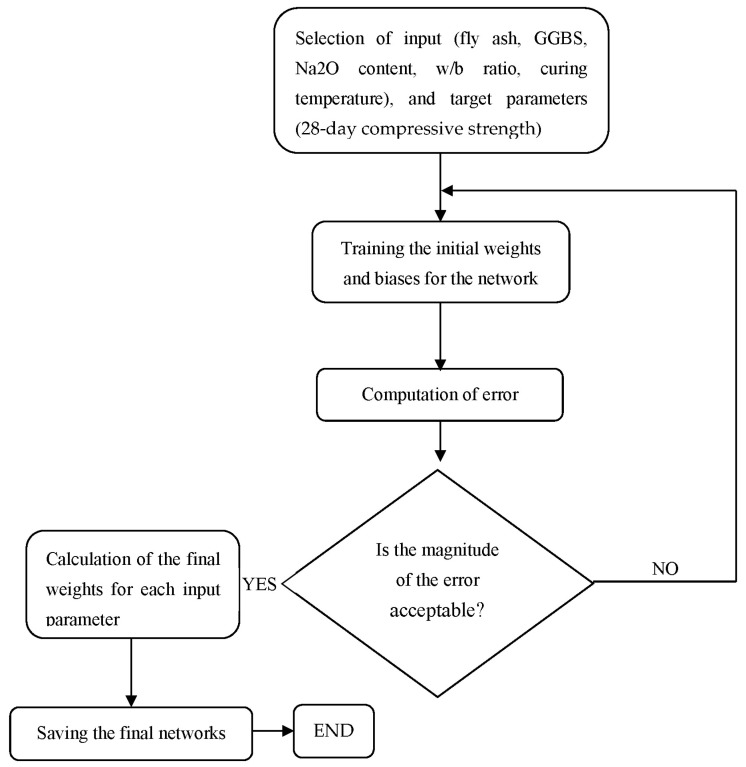
Methodology of the development of an efficient ANN model.

Overall, using multiple key phases, as shown in Figure 6, the ANN was used to forecast the 28-day compressive strength of developed geopolymer mixes. In the beginning, the data were split into two groups with a 7:3 ratio, with 70% of the data used to create the training dataset and the remaining 30% used to create the testing & validation dataset. Second, the ANN model was constructed using a training dataset (two hidden layers). Third, in order to validate and assess the effectiveness of the suggested ANN model, the projected outcomes were contrasted with the experimental data using several metrics, including mean absolute error (MAE), root mean square error (RMSE), and coefficient of determination (R^2^).

### 5.3. Multiple Linear Regression Model (MLR)

Determining the connection between two or more variables is a common task in engineering. Regression analysis is one of the effective statistical techniques that has consistently piqued scientists’ interest in this field. Regression modeling is generally thought of as the process of fitting models to data. Linear predictor functions are generated in a linear regression model to determine the relationship between the input/output parameters. It is important to note that several input variables are often used in regression analysis applications, creating “multiple linear regression” or MLR functions. In this instance, MLR analyzes the observed data and fits it into a linear equation to determine the correlation between two or more input variables. Multiple linear regression involves summarizing data and investigating the relationship between variables [40,41]. The general formula for multiple regression models is given in Equation (7) below, where *Y* is a dependent variable, *β*_0_ is a constant, and *β_j_* is a regression coefficient (*i* = 1, 2,…, *n*).

(7) $$Y=\beta_{0}+\sum_{i=1}^{n}\beta_{i}X_{i}$$

Simple regression analyses use only one independent variable (*X_j_*), while multiple regression analyses use two or more variables [42]. It is worth noting that if a data point lies on the fitted line entirely, the vertical deviation would be equal to zero. This study’s multiple linear regression model demonstrates the correlation between the one-part geopolymer paste characteristics and the experimentally measured 28-day compressive strength.

### 5.4. Informational Models Predictive

#### 5.4.1. Multiple Linear Regression Model (MLR)

The following Equation (8) expresses the most appropriate coefficients for the MLR model for estimating the compressive strength of the studied geopolymer pastes.

28-day Compressive Strength = 1.12 * (fly ash) + 1.93 * (GGBS) + 8.02 * (Na_2_O content) − 76.03 * (water/binder ratio) − 0.38 * (curing temperature) − 99.23 (8)

Concerning the above equation, the value of *R*^2^ was calculated as 0.61. This value demonstrates that MLR could not provide a sufficiently accurate approximation of the compressive strength of the studied geopolymer paste.

Stepwise regression is a statistical technique that is commonly used to identify the most important variables in a regression model. In stepwise regression, variables are added or removed from the model one at a time, based on their statistical significance, as measured by the *p*-value, until a final model that includes only the most important variables is reached. In stepwise regression analysis, variables are added or removed based on their *p*-values, with the threshold typically set at a significance level of 0.05. Variables with *p*-values less than 0.05 are considered significant and are added to the model, while variables with *p*-values greater than 0.05 are considered not significant and are removed from the model. This process is repeated until no more variables can be added or removed, resulting in a final model with a set of significant predictor variables.

In order to validate or reject the MLR analysis, a stepwise regression was performed in the study. After conducting the stepwise analysis and removing the variables fly ash, water/binder ratio, and curing temperature step-by-step, as they had high *p*-values, the following equation was obtained:

28-day Compressive Strength = (GGBS × 20.62) + (Na_2_O content × 37.99) − 15.96 (9)

**Figure 7:**
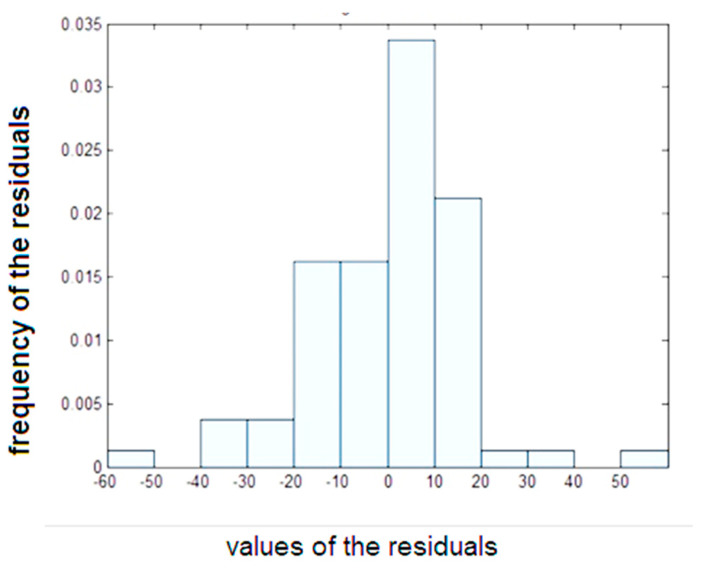
Histogram of residuals of the stepwise regression.

In this equation, the *p*-value is less than 0.05, indicating that the coefficients are satisfactory. However, the value of R-square and the RMSE was calculated at 0.563 and 16.8, respectively, indicating that the MLR analysis method unable to capture the underlying mechanism of the data. Figure 7 also shows the histogram of residuals of the stepwise regression performed in this study, demonstrating the frequency distribution of the differences between the predicted values and the actual values of the dependent variable. Residuals are the differences between the observed values of the dependent variable and the predicted values based on the regression equation. The histogram of residuals is used to evaluate the assumption of the normality of residuals. The horizontal axis of the histogram of residuals represents the values of the residuals, which are the differences between the predicted values and the actual values of the dependent variable. These residuals are typically standardized, with a mean of 0 and a standard deviation of 1, making it easier to compare residuals across different models or datasets. The vertical axis of the histogram represents the frequency of the residuals at each value. This frequency represents the number of data points that have a residual in the corresponding range. The height of each bar in the histogram indicates the number of data points with a residual value in that particular range.

Figure 7 is unsymmetric and not bell-shaped, indicating that the residuals are not normally distributed. The pattern of residuals is also irregular that confirm the model may not be appropriate for the data or that there may be some violation of assumptions.

#### 5.4.2. Artificial Neural Network

**Figure 8:**
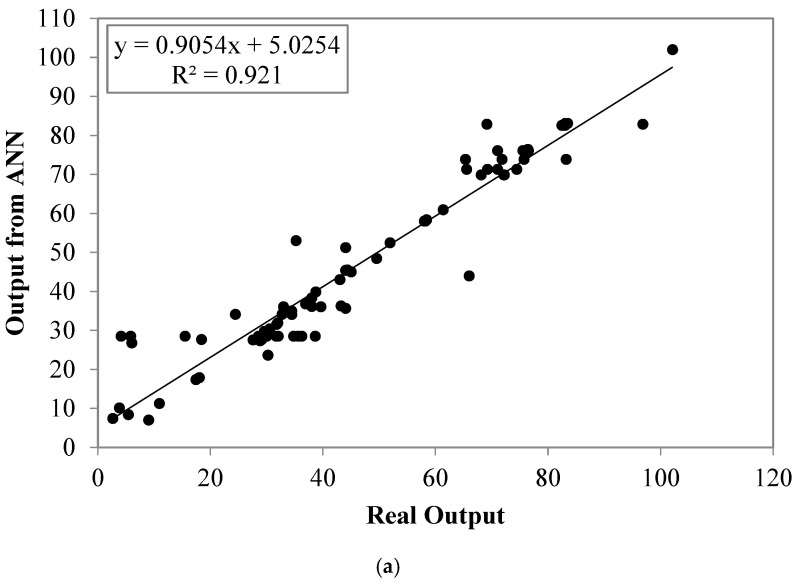

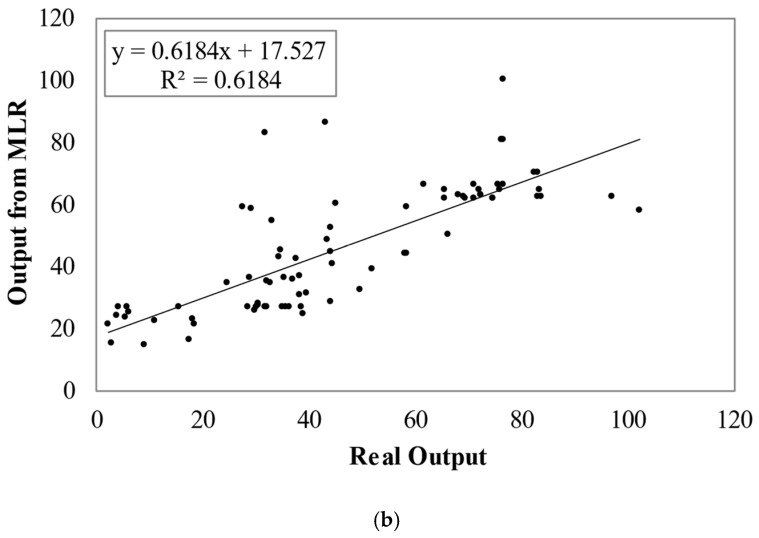
Comparison between experimental and theoretical models for compressive strength: (**a**) ANN model and (**b**) MLR model.

Figure 8 compares the actual experimental data, predictions of the ANN computational intelligence model developed in this study, and the MLR model. Upon examination of Figure 6, it became evident that the ANN model provided more reliable estimates of compressive strength when compared to the MLR model. Specifically, the ANN model appeared to provide more accurate and precise predictions of compressive strength, as evidenced by the closer alignment of the predicted values with the actual experimental data.

To further assess the performance of the two models, the authors also conducted a statistical analysis and presented the results in Table 5. The statistical analysis included various performance measures, such as mean absolute error, root mean square error, and coefficient of determination. These measures determined that the ANN model exhibited excellent agreement with the actual experimental data, while the MLR model showed comparatively lower performance.

**Figure 9:**
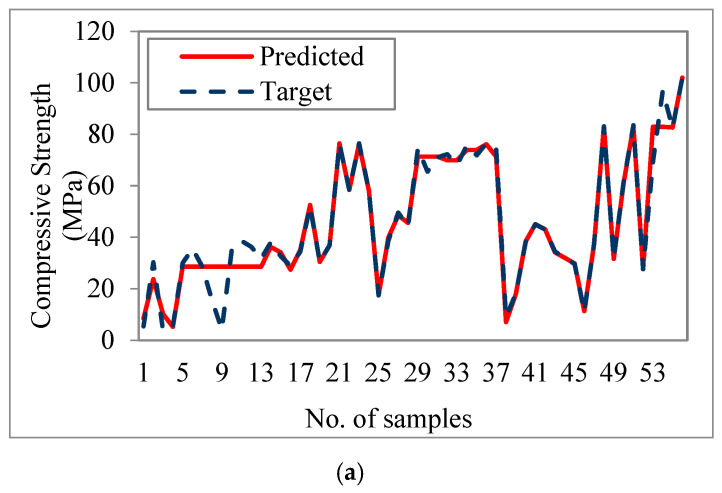

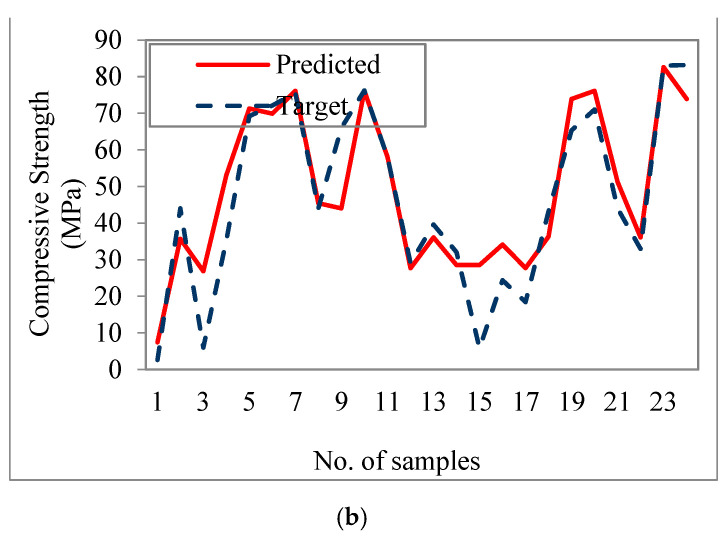
Target and predicted values of 28-day compressive strength, (**a**) training, (**b**) testing & validation.

The experimental data (represented by the discontinuous blue line) and the predicted values (represented by the continuous red line) from the training and testing data of the ANN algorithms are shown in Figure 9. The predicted compressive strength of the ANN model matched well with the target values, which is supported by both the training part (70% of data) and the validation/testing part (30% of data) for the ANN algorithms.

The following equation developed by the ANN model (extracted from MATLAB) can be employed to estimate the compressive strength of one-part geopolymer pastes.

Compressive strength = 1.16 * (fly ash) + 1.98 * (GGBS) + 0.81 * (Na_2_O content) − 0.25 * (w/b ratio) − 0.08 * (curing temperature) − 1.13 (10)

## 6. Multi-Objective Optimization Using CPLEX Tool

The development of an optimal predictive model is accomplished by: (i) establishing a model for predictions of the compressive strength of one-part geopolymer pastes using multiple linear regression (MLR) and artificial neural network (ANN), (ii) formulating the compressive strength using the best MLR’s and ANN’s models, and (iii) optimization of the formulated model to identify an optimal (maximum) value for the compressive strength.

Due to advancements in computer technology, the development of efficient algorithms and their application, as well as mathematical progress, many effective solutions have been found for Mixed Integer Programming (MIP) problems. IBM ILOG CPLEX can be used to address mathematical programming problems that require some or all variables to have integer values. These problems are known as MIPs, as the objective function and constraints may involve continuous and discrete variables, such as integers. The CPLEX Optimizer tool can generally solve linear optimization problems (LP), problems with a quadratic objective (QP), and problems with quadratic constraints (QCP). Mixed Integer Linear Programs (MILPs) refer to MIPs with linear objectives, while Mixed Integer Quadratic Programs (MIQPs) refer to MIPs with quadratic objective terms.

CPLEX provides a range of optimizers for solving linear programming problems, which can be accessed through its Con-cert Technology, Callable Library, or Interactive Optimizer. The LP problems are expressed in a certain format [43]:

(11) $$\begin{array}{c} \mathrm{Maximize}\;(\mathrm{or}\;\mathrm{Minimize})\;\sum_{i=1}^{n}C_{i}X_{i}\\ \mathrm{Subject}\;\mathrm{to}\;a_{11}\;X_{1}+a_{12}\;X_{2}+\ldots +a_{1n}\;X_{n}\;\sim \;b_{1}\\ a_{21}\;X_{1}+a_{22}\;X_{2}+\ldots +a_{2n}\;X_{n}\;\sim \;b_{2}\\ \ldots \\ a_{m1}\;X_{1}+a_{m2}\;X_{2}+\ldots +a_{mn}\;X_{n}\;\sim \;b_{m}\\ \mathrm{With}\;\mathrm{these}\;\mathrm{bounds}\;l_{1}\leq X_{1}\leq u_{1}\;\ldots \;l_{n}\leq X_{n}\leq u_{n} \end{array}$$

where the relation ~ may be greater than or equal to, less than or equal to, or simply equal to, the upper bounds *u_i_*, and lower bounds *l_i_* may be positive infinity, negative infinity, or any real number.

When a linear optimization problem is stated in that conventional form, its coefficients and values are customarily referred to by these terms: Objective function: *c*_1_, …, *c_n_*, coefficients constraint coefficients: *a*_11_, …, *a_mn_*, right-hand side: *b*_1_, …, *b_m_*, upper bounds: *u*_1_, …, *u_n_*, lower bounds: *l*_1_, …, *l_n_*, Variables or unknowns: *x*_1_, …, *x_n_*.

The variables of the objective function in the simplest linear optimization problem are mathematically continuous, meaning that there are no gaps between actual values. CPLEX implements optimizers based on simplex algorithms (both primal and dual simplex), primal-dual logarithmic barrier algorithms, and a sifting technique to resolve such linear programming issues. Computations for an experimental study of the mathematical model proposed in this study were carried out on a personal computer with AMD Ryzen 7 2700X Eight-Core Processor 3.70 GHz having a Windows 10 operating system with 16 GB RAM. The Multi-Objective MIP was conducted on CPLEX 12.9, and the optimal (maximum) value of compressive strength for the geopolymer mixture design was obtained. The decision variables are considered integer values. The objective function and related constraints are as follows:

*Decision* variables

(12) $$\mathrm{Compressive}\;\mathrm{strength}:\;\max\;\sum_{i=1}^{n}C_{i}X_{i}=(1.16*\mathrm{fly}\;\mathrm{ash})+(1.98*\mathrm{GGBS})+(0.81*{\mathrm{Na}}_{2}O)+(-0.25*\mathrm{WB})+\;(-0.08*\mathrm{curing}\;\mathrm{temperature})-1.13$$

Constraints

15 ≤ fly ash ≤ 60,(13)

20 ≤ GGBS ≤ 70,(14)

Fly ash + GGBS = 100,(15)

0.30 ≤ w/b ratio ≤ 0.40,(16)

Fly ash ≤ GGBS,(17)

1.50 ≤ Na_2_O ≤ 12.50, (18)

20 ≤ curing temperature ≤ 60,(19)

The objective of Equation (12) is to maximize the compressive strength of the one-part geopolymer paste mixture. The constraint set by Equation (13) assigns fly ash from 15 to 60%, and the constraint specified by Equation (14) sets GGBS between 20 and 70%. Meanwhile, the constraint set by Equation (15) is defined simply because the cumulative values of fly ash and GGBS must be equivalent to 100%. For this optimization, the water/binder ratio value was selected between 0.30 (minimum) and 0.40 (maximum), as shown by Equation (16). The minimum values of Na_2_O and curing temperature were set as 1.5% and 20 °C, while the maximum values were selected as 12.50% and 60 °C, respectively, as shown by Equations (18) and (19).

Solving the mathematical model using CPLEX concerning the set constraints provides the optimal mixture having maximum compressive strength. Concerning all parameters, objective functions, and constraints, the CPLEX calculates the maximum compressive strength value as 72.35 MPa, where the optimal values of each input parameter are shown in Table 6.

## 7. Sensitivity Analysis

The previous section established the optimal values of FA, GGBS, Na_2_O content, w/b ratio, and curing temperature for the ANN model to attain the highest compressive strength of the one-part geopolymer paste. This section focuses on performing a sensitivity analysis to assess the influence of the input parameters on the 28-day compressive strength. Through this analysis, the extent to which the output of the model can be affected by changes in the input parameters is determined [44]. In general, there are two main categories of sensitivity analysis: local sensitivity analysis (LSA) and global sensitivity analysis (GSA).

Local sensitivity analysis (LSA) is a method used to evaluate the sensitivity of a model’s output to small perturbations in the input parameters around a specific point. It helps to identify which input parameters have the most significant impact on the model’s output at a specific point in the input space. On the other hand, global sensitivity analysis (GSA) examines the sensitivity of a model’s output to variations in input parameters across the entire parameter space. It is used to identify which input parameters are the most influential over a wide range of inputs and determine how these parameters affect the model’s output. Both LSA and GSA are important tools for understanding the behavior of a model and can be used to inform model calibration, parameter estimation, and uncertainty quantification. Local sensitivity analysis (LSA) is based on the first-order derivative of the model output with respect to the input parameters. Global sensitivity analysis (GSA) is based on the variance decomposition of the model output with respect to the input parameters.

In general, local sensitivity analysis (LSA) is concerned with analyzing the effects of individual input variables on the overall system performance, while global sensitivity analysis (GSA) examines the impact of input variables across their entire range of values and assesses the uncertainty in system performance due to interactions between variables or when variables are varied independently. Due to the nonlinear nature of the current study, GSA was deemed more appropriate for evaluating the influence of input variables on overall system performance [45].

**Figure 10:**
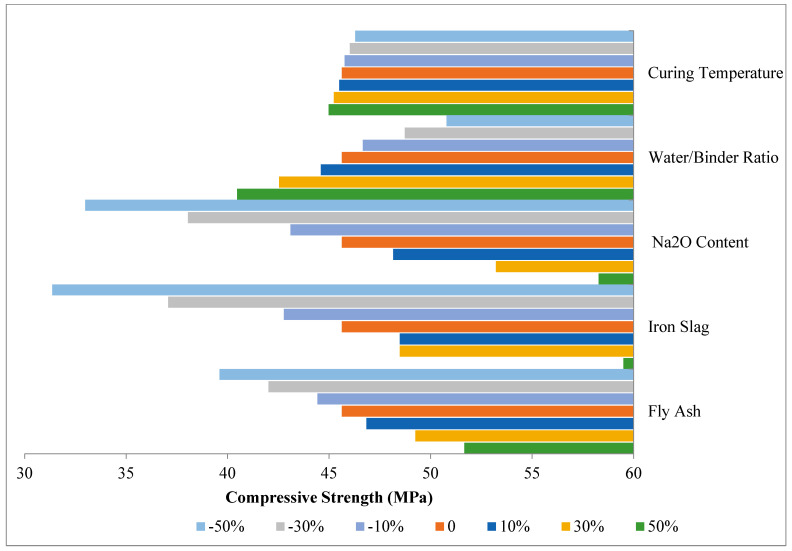
Sensitivity analysis of variables for compressive strength of one-part geopolymer paste.

The sensitivity analysis is shown in Figure 10. The results indicate that the Na_2_O and GGBS contents significantly influenced the 28-day compressive strength. For instance, decreasing the GGBS by 50% led to a sharp decrease in compressive strength, estimated at around 31 MPa, while a significant increase in compressive strength of 59.5 MPa was estimated for a 50% increase in GGBS. The Na_2_O content also played an important role in compressive strength; the larger the Na_2_O content, the higher the compressive strength. On the other hand, a lower water/binder ratio led to higher compressive strength, as expected.

## 8. Concluding Remarks

In this research, by compiling 80 different mixtures of one-part geopolymer pastes, the effects of constituent materials, Na_2_O content of the alkaline sources, curing condition, and water/binder ratio on the 28-day compressive strength were examined in detail. ANN model using the Levenberg–Marquardt algorithm was also developed to estimate the compressive strength and its sensitivity to the input variables. Using the weights and biases of the ANN model, a CPLEX-based optimization methodology was developed to optimize the binder constituent and alkaline sources to achieve the highest compressive strength. Based on this work, the following observations and conclusions can be drawn:

- The results confirm that there is a direct relationship between the GGBS and Ca_2_O content and the 28-day compressive strength. The sensitivity analysis confirmed that a 50% decrease in GGBS content leads to an estimated compressive strength of around 31 MPa, while a 50% increase leads to an estimated compressive strength of 59.5 MPa. The Na_2_O content also strongly influences compressive strength, with higher Na_2_O content resulting in higher strength. Lower water-to-binder ratios are also associated with higher compressive strength, consistent with expectations.
- The R^2^ value of the MLR model is 0.61, where the coefficients of the variables in the equation show their relative contribution to the compressive strength, with Na_2_O content having the highest coefficient. The negative coefficients for water/binder ratio indicate that increasing this variable decreases the compressive strength. However, the model is not accurate enough to provide a precise estimation of the compressive strength of the paste.
- The proposed ANN model can adequately estimate the compressive strength of fly ash slag-based one-part geopolymer paste (with *R*^2^ = 0.94 and RMSE = 0.07). Such an assessment of the relative significance of various features and their impact on compressive strength can assist material scientists/designers in making reliable decisions about the source materials to use for achieving the required strength performance.
- The mathematical model was solved using CPLEX with set constraints to determine the optimal mixture for maximum compressive strength. The resulting optimal input parameters to achieve the maximum compressive strength value of 72.35 MPa were calculated fly ash at 12.30%, GGBS at 21.6%, Na2O content at 12.50%, water/binder ratio at 0.3, and curing temperature at 20 °C.
- The developed CPLEX mathematical model can be employed as a reliable tool for preparing geopolymer past with optimal mixture design and compressive strength. This technique can lead to an efficient input variable selection and a reduction in training time without compromising model accuracy.
- The shortcomings of this research include a limited sample size, a narrow range of input parameters, and reliance on the specific type of binders (fly ash and GGBS). To address these issues, future research could involve a larger and more diverse sample size, a wider range of input parameters, and the use of various types of binders and alkaline sources. Additionally, incorporating more advanced machine learning techniques, such as deep learning, could enhance the accuracy of the predictive model and its optimization.

## Figures and Tables

**Table 1:** Studied mixture design and their 28-day compressive strength.

Mix No	Reference	Fly Ash (%)	GGBS (%)	Activator Na_2_O (%)	Water/Binder Ratio	Curing Temperature (°C)	28-d Compressive Strength (MPa)
1	[17]	100	0	4.25	0.3	23	2.63
2		90	10	4.22	0.3	23	5.4
3		85	15	4.21	0.3	23	30.2
4		80	20	4.21	0.3	23	39.6
5		85	15	3.78	0.3	23	3.8
6		85	15	3.48	0.3	23	2
7	[18]	85	15	4	0.28	20	44
8		85	15	4	0.3	20	30
9		85	15	4	0.32	20	6
10		85	15	4	0.3	20	32.02
11		85	15	4	0.3	20	35.54
12		85	15	4	0.3	20	28.52
13	[19]	85	15	4	0.3	20	15.48
14		85	15	4	0.3	20	5.791
15		85	15	4	0.3	20	4.11
16		85	15	4	0.3	20	34.77
17	[20]	85	15	4	0.3	20	38.6
18		85	15	4	0.3	20	36.22
19		85	15	4	0.3	20	31.66
20		80	20	4	0.3	20	38
21		80	20	4	0.25	20	24.4
22		80	20	4	0.25	20	32.7
23	[21]	80	20	4	0.23	20	28.8
24		70	30	4	0.25	20	34.4
25		70	30	4	0.3	20	51.9
26		75	25	5.1	0.394	60	18.4
27	[22]	75	25	6	0.4	60	30.5
28		75	25	6	0.3	60	36.9
29	[23]	50	50	4	0.35	60	35.2
30		0	100	5	0.35	20	76.4
31		50	50	5	0.35	20	58.4
32	[24]	0	90	5	0.35	20	76.4
33		45	45	5	0.35	20	58.2
34		100	0	4	0.25	25	17.4
35		100	0	5	0.25	25	38.7
36		100	0	6	0.25	25	49.5
37		100	0	7	0.25	25	44.3
38	[25]	100	0	8	0.25	25	43.2
39		50	50	6	0.4	25	74.4
40		50	50	6	0.4	25	65.5
41		50	50	6	0.4	25	71
42		50	50	6	0.4	25	69.2
43		50	50	6	0.38	25	72.2
44		50	50	6	0.38	25	68.1
45		50	50	6	0.38	25	72.1
46		50	50	6	0.36	25	75.7
47		50	50	6	0.36	25	65.3
48		50	50	6	0.36	25	71.8
49		50	50	6	0.34	25	76.5
50		50	50	6	0.34	25	75.5
51		50	50	6	0.34	25	71
52	[26]	50	50	6	0.4	25	74.4
53		100	0	4	0.282	23	9
54		90	10	4	0.282	23	18
55		70	30	4	0.313	23	38
56		60	40	4	0.319	23	44
57		50	50	4	0.324	23	44
58		40	60	4	0.325	23	45
59		0	100	4	0.412	23	43
60		0	95	1.5	0.45	20	32.96
61		0	90	1.5	0.45	20	34.44
62		0	85	1.5	0.45	20	31.95
63	[27]	0	80	1.5	0.45	20	29.56
64		100	0	5	0.25	30	10.9
65		100	0	7.5	0.25	30	37.57
66		100	0	10	0.25	30	83.03
67		100	0	12.5	0.25	30	31.74556
68		100	0	10	0.2	30	61.35
69		100	0	10	0.25	30	83.45
70	[28]	100	0	10	0.3	30	27.55
71		50	50	6	0.4	23	69.1
72		50	50	6	0.4	23	96.83
73		50	50	4.5	0.4	23	65.96
74		50	50	6	0.3	23	82.44
75		50	50	6	0.3	23	82.98
76		50	50	4.5	0.3	23	102.1
77		0	90	5	0.35	20	76.21
78		45	45	5	0.35	20	57.99
79		50	50	6	0.36	25	83.19
80		60	40	7.5	0.5	23	29.09

**Table 2:** Input/output parameters range.

Parameter	Unit	Lower Limit	Upper Limit	Average
Fly ash content	%	0	100	65.3
GGBS	%	0	100	33.56
Na_2_O dosage	%	1.5	12.5	5.13
w/b ratio	-	0.2	0.5	0.33
Curing temperature	°C	20	60	25
28-day compressive strength	MPa	2	102.1	46

**Table 3:** Physical and chemical features of binder materials reported by [20].

Physical and Chemical Features	GGBS	Fly Ash
Specific gravity	2.9	2.2
Aver. particle size (μm)	12.8	10
SiO_2_	30.8	57.20
Al_2_O_3_	10.9	28.81
Fe_2_O_3_	0.64	3.67
CaO	51.8	5.16
MgO	4.57	1.48
K_2_O	0.36	0.94
Na_2_O	0.45	0.08
SO_3_	0.06	0.10
LOI	0.22	0.12

**Table 4:** Weights and biases for the selected ANN model.

Output	Weights					Biases
	Fly Ash	GGBS	Na_2_O Content	w/b Ratio	Curing Temperature	
Compressive strength	1.16	1.98	0.81	−0.25	−0.08	−1.13

**Table 5:** Evaluation of statistical matrices of ANN and MLR models.

Model	Statistical Matrices			
	*R* ^2^	MSE	RMSE	MAE
MLR	0.61	236.21	15.37	11.63
ANN	0.92	0.0049	0.07	0.041

**Table 6:** Optimal input parameter values to achieve maximum compressive strength.

Max: CS	Fly Ash (%)	GGBS (%)	Na_2_O Content (%)	w/b Ratio	Curing Temperature
74.80	12.30	21.65	12.50	0.30	20

## Data Availability

The dataset we have used in this research is available in Table 1 of the manuscript.
